# Comparative transcriptome analysis reveals the importance of phenylpropanoid biosynthesis for the induced resistance of 84K poplar to anthracnose

**DOI:** 10.1186/s12864-024-10209-1

**Published:** 2024-03-22

**Authors:** Fei Xing, Linxuan Zhang, Wei Ge, Haixia Fan, Chengming Tian, Fanli Meng

**Affiliations:** 1 The Key Laboratory for Silviculture and Conservation of Ministry of Education, College of Forestry, Beijing Forestry University, 100083 Beijing, China; 2 Beijing Key Laboratory for Forest Pest Control, College of Forestry, Beijing Forestry University, 100083 Beijing, China

**Keywords:** 84K poplar, *Colletotrichum gloeosporioides*, Induced resistance, Phenylpropanoid biosynthesis

## Abstract

**Background:**

Poplar anthracnose, which is one of the most important tree diseases, is primarily caused by *Colletotrichum gloeosporioides*, which has been detected in poplar plantations in China and is responsible for serious economic losses. The characteristics of 84K poplar that have made it one of the typical woody model plants used for investigating stress resistance include its rapid growth, simple reproduction, and adaptability.

**Results:**

In this study, we found that the resistance of 84K poplar to anthracnose varied considerably depending on how the samples were inoculated of the two seedlings in each tissue culture bottle, one (84K-Cg) was inoculated for 6 days, whereas the 84K-DCg samples were another seedling inoculated at the 6th day and incubated for another 6 days under the same conditions. It was showed that the average anthracnose spot diameter on 84K-Cg and 84K-DCg leaves was 1.23 ± 0.0577 cm and 0.67 ± 0.1154 cm, respectively. Based on the transcriptome sequencing analysis, it was indicated that the upregulated phenylpropanoid biosynthesis-related genes in 84K poplar infected with *C. gloeosporioides*, including genes encoding PAL, C4H, 4CL, HCT, CCR, COMT, F5H, and CAD, are also involved in other KEGG pathways (i.e., flavonoid biosynthesis and phenylalanine metabolism). The expression levels of these genes were lowest in 84K-Cg and highest in 84K-DCg.

**Conclusions:**

It was found that PAL-related genes may be crucial for the induced resistance of 84K poplar to anthracnose, which enriched in the phenylpropanoid biosynthesis. These results will provide the basis for future research conducted to verify the contribution of phenylpropanoid biosynthesis to induced resistance and explore plant immune resistance-related signals that may regulate plant defense capabilities, which may provide valuable insights relevant to the development of effective and environmentally friendly methods for controlling poplar anthracnose.

**Supplementary Information:**

The online version contains supplementary material available at 10.1186/s12864-024-10209-1.

## Background

As one of the three important fast-growing tree species, poplar is crucial for the construction of protection forests, while also serving as a source of industrial timber. China contains the largest poplar plantation area, which now exceeds 8.5 million hectares [1]. With the expansion of the poplar planting area worldwide, poplar diseases have become a major concern in the forestry industry and among ecologists [2]. During growth, poplar trees are susceptible to multiple pathogens and pests. Some of these pathogens are responsible for anthracnose, which is the most severe poplar disease that causes extensive damage to the leaves. Previous studies on poplar anthracnose identified the causative pathogens as *Colletotrichum* species, including *C. graminicola* [3], *C. gloeosporioides*, and *C. poplar* [4], of which *C. gloeosporioides* is the main pathogen causing poplar anthracnose [3, 4]. Hence, *C. gloeosporioides* was analyzed in this study. The notable characteristics of the poplar hybrid (*Populus alba* × *Populus glandulosa*) designated as 84K include its rapid growth, relatively simple reproduction, and adaptability, which may help to explain why it is increasingly being used as one of the typical woody model plants for stress resistance research.

The interaction between plants and surrounding organisms, including microorganisms, is a common phenomenon. The long-term co-evolution of plants and microorganisms resulted in the development of adaptive mechanisms enabling plants to withstand microbial infections [5]. These mechanisms facilitate the recognition of microbial signaling molecules and the activation of specific physiological responses. Secondary metabolites are the ultimate manifestation of microbe-induced changes in gene and protein expression, with the types and contents of secondary metabolites reflecting the physiological and pathological status of plants [1].

There are two common mechanisms mediating plant disease resistance, namely rapid local reactions (e.g., hypersensitive response) and systemic acquired resistance [6]. Induced resistance involves several physiological and biochemical reactions that occur in plants after certain pathogen-related stimuli are perceived. Examples include increases in the contents of soluble carbohydrates and phenolic compounds, the production and accumulation of plant defense hormones, changes in various enzyme activities, and the production of pathogenesis-related proteins, ultimately leading to increased resistance to pathogens [1]. Phenylalanine ammonia lyase (PAL) is a key enzyme for the synthesis of phytoalexins, lignin, and phenols. When plants are induced, PAL activity increases significantly, thereby affecting phenylpropanoid biosynthesis [7].

Phenylpropanoid biosynthesis is an important secondary metabolic pathway in plants, with the metabolites produced by this pathway accounting for approximately 20% of the secondary metabolites in plants. The molecular basis of phenylpropanoid biosynthesis and the mechanism precisely regulating metabolite biosynthesis have been studied extensively in recent decades [8]. Structural genes and transcription factor genes in the phenylpropanoid biosynthesis pathway have been cloned and functionally verified in a variety of plants [1, 9]. However, the effects of the phenylpropanoid biosynthesis pathway on induced resistance in poplar have not been thoroughly characterized. Most of the existing studies on poplar anthracnose mainly focused on how *C. gloeosporioides* infects poplar, the interaction between the metabolic pathway and *C. gloeosporioides*, and the changes in resistance-related compounds in different poplar species. Unfortunately, the infection of poplar by *C. gloeosporioides* has rarely been explored from the perspective of the poplar tree [1, 10, 11].

In this study, we analyzed the induced resistance of 84K poplar to anthracnose by comparing the poplar samples under different conditions. The results of this study may provide new insights into the molecular basis of the induced resistance of 84K poplar to anthracnose.

## Methods

### Plant and fungal materials

Tissue-cultured 84K poplar seedlings and *C. gloeosporioides* strain CFCC 80,308 (Cg) were provided by the Forest Pathology Laboratory of Beijing Forestry University [12]. Two 84K poplar seedlings were cultivated in one tissue culture bottle at 25 ± 2°C with a 16-h light (2,000 lx):8-h dark cycle and 60–70% relative humidity. The seedlings were grown until they had six leaves and then they were inoculated with *C. gloeosporioides* (Figure S1).

After *C. gloeosporioides* was grown on potato dextrose agar medium for 7 days, sterile water was repeatedly added to cover the medium surface and then collected. The concentration of the resulting spore (conidia) solution was adjusted to 10^6^/mL, after which 30 µL was used to inoculate the 84K poplar seedlings. The control 84K poplar seedlings were treated with 30 µL sterile water (i.e., 84K-No samples). Of the two seedlings in each tissue culture bottle, one (84K-Cg) treated with 30 µL spore (conidia) solution was inoculated for 6 days, whereas the 84K-DCg sample treated with 30 µL spore (conidia) solution was another seedling inoculated at the 6th day and incubated for another 6 days (the 12th day) under the same conditions. The follow-up experiments were conducted using the three 84K inoculated seedlings (i.e., 84K-No, 84K-Cg, and 84K-DCg samples) (Figure S1). Meanwhile, the seedling symptoms were based on the size of lesion on the 6th inoculation day. The experiment was completed using three biological replicates and five technical replicates.

### Total RNA extraction

Total RNA was extracted from the 84K poplar samples (whole plants) using the RNAprep Pure Plant kit (Tiangen, Beijing, China) and then the RNA integrity was evaluated using the Agilent 2100 Bioanalyzer. For the transcriptome sequencing analysis, cDNA libraries were prepared using the Illumina Kit (Illumina, San Diego, CA, USA) and then sequenced on the Illumina NovaSeq 6000 platform. Trimmomatic v.0.33 (RWTH Aachen University, Aachen, Germany) was used to screen for quality and filter the original Illumina reads to remove low-quality reads. The *Populus alba* × *Populus tremula* genome was selected as the reference genome (https://db.cngb.org/search/project/CNP0000339/).

### Assembly and quality evaluation

The transcriptome analysis was performed using three biological replicates. Trinity (2.0.6) was used to assemble clean reads (after removing PCR duplicates to increase the assembly efficiency) and then Tgicl (2.1) was used to cluster and eliminate redundant transcripts to obtain unigenes. Next, Tgicl was used to cluster and remove redundant high-quality unigenes from each sample to obtain non-scalable and non-redundant unigenes for the subsequent analysis. The integrity and quality of the assembled transcriptome were evaluated using conserved sequences from the Benchmarking Universal Single-Copy Orthologs database.

### Gene expression analysis

Bowtie2 (2.2.5) was used to map the clean reads of each sample to the assembled transcriptome. According to the comparison results, RSEM (1.2.8) was used to quantify the mapped reads for each sample and determine the expression level of each gene in nine poplar samples. The gene expression levels were standardized according to fragments per kilobase of exon model per million mapped reads (FPKM) values. On the basis of a coefficient of variation threshold of ≤ 0.8, three biological replicates were screened for genes with highly correlated expression levels.

### Functional characterization of unigenes

To functionally annotate all unigenes, BLASTX (Basic Local Alignment Search Tool) was used to compare the transcripts with sequences in public databases (E-value ≤ 10^− 5^ set as the threshold for significance). The following databases were analyzed: Nr (http://www.ncbi.nlm.nih.gov), Swiss-Prot (http://www.ncbi.nlm.nih.gov), COG (http://www.ncbi.nlm.nih.gov/COG), and KEGG (http://www.genome.jp/kegg). Additionally, on the basis of the Nr annotations, Blast2GO (2.5.0) was used to assign GO terms to all unigenes (https://www.biocloud.net).

### Identification of differentially expressed genes

Pairwise comparisons of different treatments were performed and the differentially expressed genes (DEGs) were identified using DEseq2 and the following criteria:|log_2_(fold-change)| ≥ 1 and *p* ≤ 0.1. A hierarchical clustering analysis of the DEGs was performed using the pheatmap function in the R software. According to the GO annotation results, KEGG annotation results, and official classifications, the DEGs were functionally annotated and an enrichment analysis was conducted using the phyper function in R (4.0.3). An FDR correction was performed for the *p* values. Functions with a Q value ≤ 0.05 were considered significantly enriched.

### Quantitative real-time PCR (qRT-PCR)

The expression of key DEGs was analyzed by qRT-PCR. Total RNA was reverse transcribed using the Hifair® II 1st Strand cDNA Synthesis SuperMix for qPCR (with gDNA digester plus) (Yeasen Biotechnology, Shanghai, China). The primer pairs (Table S1) for the candidate genes were designed using Primer Premier 6.0. The qRT-PCR analysis was performed using the CFX Connect Real-Time PCR instrument (Bio-Rad, USA) and the Hieff UNICON® Universal Blue qPCR SYBR Green Master Mix (Yeasen Biotech-nology). The *18 S* gene served as an internal reference in this study. Relative gene expression was calculated using the 2^−∆∆Ct^ method [13].

### Phenylpropanoid and flavonoid extraction and analyses by HPLC-MS/MS

Samples from different treatments (84K-No, 84K-Cg, and 84K-DCg) were lyophilized at −40°C for 24 h and then stored in an ultra-low temperature freezer. Frozen samples were ground to a powder (30 Hz, 1 min) using a MM 400 grinder (Retsch, Haan, Germany). The ground material was stored at −20°C until analysis. The analysis methods of high performance liquid chromatography-tandem mass spectrometry (HPLC-MS/MS) were as described in Zhang et al. [1]. For the qualitative analysis of phenylpropanoid and flavonoid metabolites, the type and relative content of each compound was determined from HPLC chromatograms.

### Data analysis

The generated data were statistically analyzed using SPSS 17.0 (SPSS Co., Ltd., Chicago, Illinois, USA) and Origin 8.0 (Origin Lab, Northampton, Massachusetts, USA). A one-way analysis of variance involving Duncan’s test was used to determine the significance (*p* < 0.05) of the differences between treatments.

### Data deposition

All transcriptome data have been deposited in National Center for Biotechnology Information (NCBI) under accession codes of PRJNA 1,052,950.

## Results

### Disease symptoms of the 84K poplar seedlings under different conditions

**Fig. 1:**
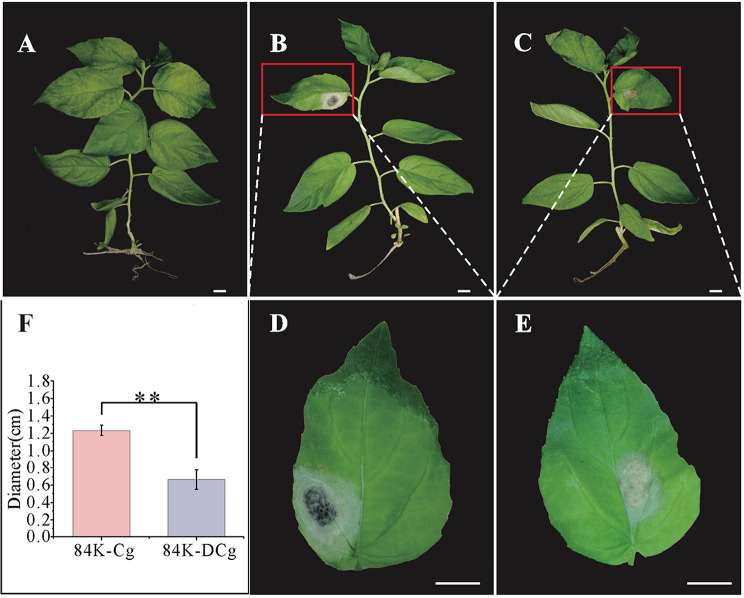
Differences in the resistance of 84K poplar seedlings to anthracnose. (**A**) 84K-No. (**B**) 84K-Cg. (**C**) 84K-DCg. (**D**) Enlarged 84K-Cg leaf. (**E**) Enlarged 84K-DCg leaf. (**F**) Average leaf lesion diameter. Error bars represent the standard error of the mean; **p < 0.01. The scale bars represent 1 cm

After 84K poplar seedlings (Fig. 1A) were inoculated with *C. gloeosporioides*, the leaf lesion area was examined for 6 consecutive days. The lesion size on the 84K-Cg leaf blades expanded rapidly (Fig. 1B and D). In contrast, the lesion area on the 84K-DCg leaf blades expanded slowly and the blackening of the lesion was less extensive (Fig. 1C and E). The average anthracnose spot diameter on the 84K-Cg and 84K-DCg leaves at 6 days post-inoculation was 1.23 ± 0.0577 cm and 0.67 ± 0.1154 cm, respectively; this difference was significant (Fig. 1F), which were measured at the 6th inoculation day.

### Transcriptome sequencing and data assembly

We sequenced the transcriptomes of the 84K-No, 84K-Cg, and 84K-DCg samples to explore the changes in gene expression potentially related to the induced resistance of 84K poplar to anthracnose. The raw reads generated for the 84K-No, 84K-Cg, and 84K-DCg samples comprised 33,894,057 bp, 35,675,816 bp, and 35,226,466 bp, respectively (Table 1 and Fig. S2). For each sample, at least 5.92 Gb clean data were generated, with a quality score of Q30 for at least 94.29% of the data. The clean reads for each sample were mapped to the reference genome, with a mapping rate of 85.66–97.06%.

**Table 1:** Quality of the transcriptome sequencing data for the 84K-No, 84K-Cg, and 84K-DCg samples

Sample	Clean reads	Mapped reads	GC content (%)	Q30 (%)	Total mapping (%)	Uniquely mapping (%)
84K-No	33,894,057	65,515,812	45.04	94.29	96.65	87.21
84K-Cg	35,675,816	67,680,377	46.01	95.10	94.85	81.96
84K-DCg	35,226,466	68,383,385	45.08	94.50	97.06	87.28

### Differentially expressed genes identified by the transcriptome analysis

**Fig. 2:**
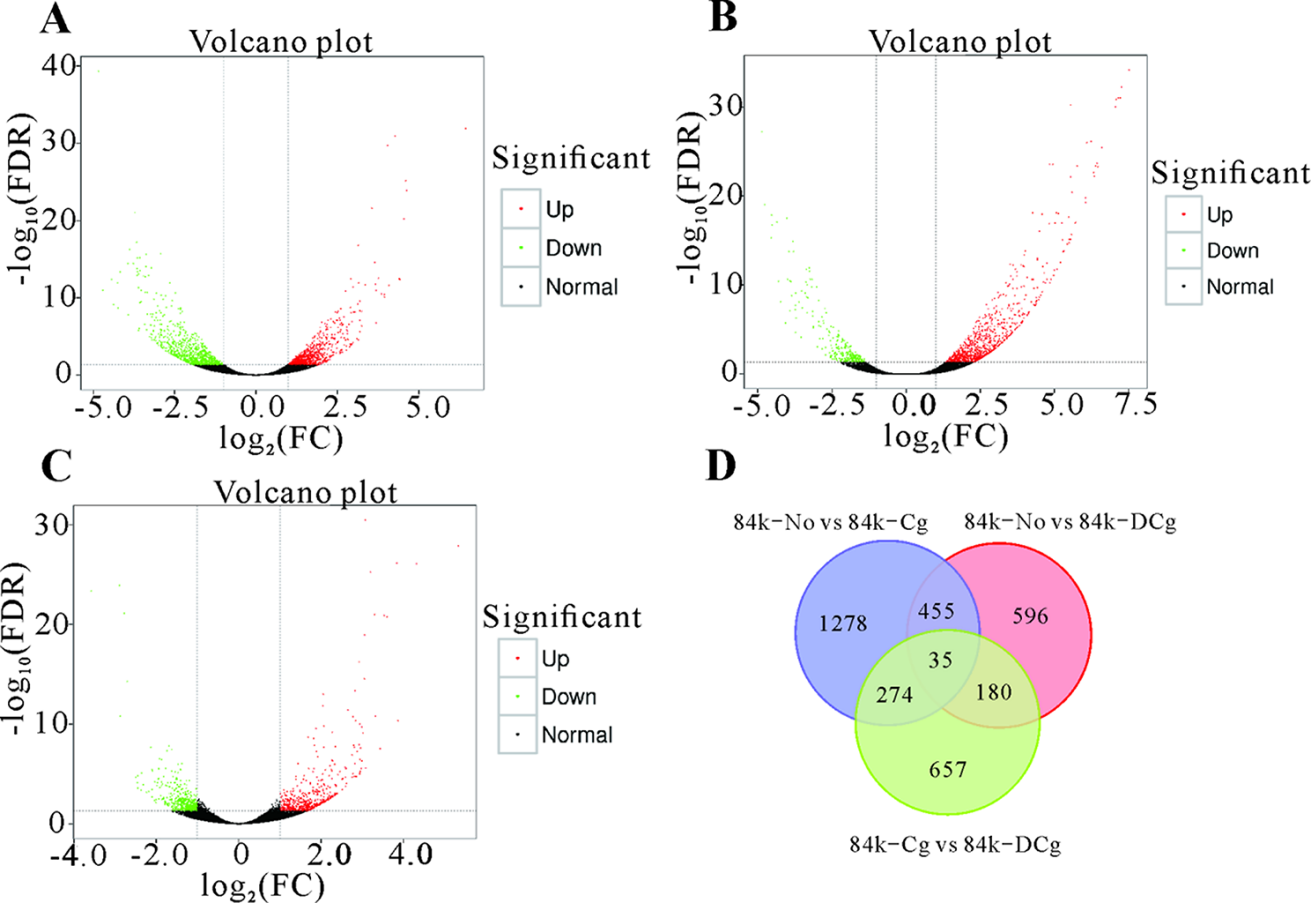
Volcano plots of the differentially expressed genes (DEGs) revealed by the (**A**) 84K-No vs. 84K-Cg, (**B**) 84K-No vs. 84K-DCg, and (**C**) 84K-Cg vs. 84K-DCg comparisons. (**D**) Venn diagram of DEGs

The sequenced transcriptomes were compared to identify the DEGs among the 84K-No, 84K-Cg, and 84K-DCg seedlings. A volcano map was produced to visualize the distribution of each group of DEGs. And we generated Venn diagrams to profile the DEG distribution among the 84K-No vs. 84K-Cg, 84K-No vs. 84K-DCg, and 84K-Cg vs. 84K-DCg. The comparison between 84K-No and 84K-Cg detected 2,042 DEGs (1,015 upregulated and 1,027 downregulated) (Fig. 2A and D). The comparison between 84K-No and 84K-DCg revealed 1,266 DEGs (969 upregulated and 297 downregulated) (Fig. 2B and D). The comparison between 84K-Cg and 84K-DCg identified 1,146 DEGs (599 upregulated and 547 downregulated) (Fig. 2C and D).

### GO analysis of DEGs

The Gene Ontology (GO)-based functional annotations indicated that molecular-function-related GO terms were shown in the Table 2. The GO terms in the molecular function category were assigned to 10 upregulated DEGs (84K-No vs. 84K-Cg, 84K-No vs. 84K-DCg, and 84K-Cg vs. 84K-DCg) related to metabolic activities, including the following: phenylpropanoid biosynthesis; flavonoid biosynthesis; phenylalanine metabolism; phenylalanine, tyrosine, and tryptophan biosynthesis; tyrosine metabolism; isoquinoline alkaloid biosynthesis; and tropane, piperidine, and pyridine alkaloid biosynthesis (Table 2). In addition, 10 downregulated DEGs (84K-No vs. 84K-Cg, 84K-No vs. 84K-DCg, and 84K-Cg vs. 84K-DCg) included genes related to galactose metabolism, carbohydrate transport and metabolism, and ABC transporters (Table 2).

**Table 2:** Top 10 upregulated and downregulated genes in the 84K-No, 84K-Cg, and 84K-DCg samples

Condition	ID	log_2_FC	*p*-value	Annoation
84K-No- 84K-Cg-up	Pop_A05G016859	6.4480	1.29 × 10^− 32^	ADP-ribosylation factor family
	Pop_G04G023258	4.6318	1.34 × 10^− 24^	Thi4 family
	Pop_A04G018288	4.6029	6.29 × 10^− 26^	Thi4 family
	Pop_UnG036059	4.5487	5.81 × 10^− 21^	Alcohol dehydrogenase
	Pop_G08G064147	4.4255	4.52 × 10^− 13^	Phosphoglucomutase
	Pop_G08G082357	4.4002	3.54 × 10^− 13^	Aconitase family
	Pop_A14G000674	4.2726	1.28 × 10^− 31^	Universal stress protein family
	Pop_A08G046037	4.0846	2.43 × 10^− 12^	Aconitase family
	Pop_G09G077661	4.0459	1.87 × 10^− 30^	RNA recognition
	Pop_UnG039873	3.9468	3.71 × 10^− 11^	TAR1 protein
84K-No- 84K-Cg-down	Pop_G12G050744	4.8319	4.82 × 10^− 40^	Calcineurin-like phosphoesterase
	Pop_A13G082693	4.7002	9.95 × 10^− 12^	Acylhydrolase
	Pop_A13G054433	4.4305	4.19 × 10^− 13^	Glycosyl transferase family 8
	Pop_G10G007231	4.3708	6.97 × 10^− 10^	Multicopper oxidase
	Pop_A01G054516	4.2456	1.19 × 10^− 12^	Thaumatin family
	Pop_A08G006905	4.2376	2.09 × 10^− 9^	Glycosyltransferase like family 2
	Pop_A19G052343	4.1705	4.08 × 10^− 14^	GDA1/CD39 family
	Pop_G08G057836	3.9297	3.77 × 10^− 15^	Ultraviolet-B receptor
	Pop_G03G011194	3.9262	1.57 × 10^− 8^	Pectinesterase
	Pop_G06G075964	3.8826	5.35 × 10^− 17^	SPX domain-containing protein 1
84K-No-84K-DCg-up	Pop_A12G074025	7.5140	6.14 × 10^− 35^	Glycosyl hydrolases family 18
	Pop_G19G028437	7.2501	5.20 × 10^− 33^	Trypsin and protease inhibitor
	Pop_A05G072069	7.2098	9.27 × 10^− 32^	Lipoxygenase
	Pop_G19G028438	7.1527	9.81 × 10^− 32^	Trypsin and protease inhibitor
	Pop_G01G013394	7.0804	1.34 × 10^− 31^	Cupin
	Pop_G07G061254	7.0531	9.34 × 10^− 31^	Trypsin and protease inhibitor
	Pop_G19G028457	6.5901	3.40 × 10^− 26^	Trypsin and protease inhibitor
	Pop_A07G070554	6.4640	4.92 × 10^− 24^	Trypsin and protease inhibitor
	Pop_A05G016859	6.4330	2.11 × 10^− 24^	ADP-ribosylation factor family
	Pop_G17G033368	6.3832	4.12 × 10^− 24^	Patatin-like phospholipase
84K-No-84K-DCg-down	Pop_G14G000556	4.8545	5.57 × 10^− 28^	FKBP-type-transisomerase
	Pop_A14G045280	4.8544	5.51 × 10^− 28^	FKBP-type-transisomerase
	Pop_A17G034520	4.7632	9.58 × 10^− 20^	Hsp90 protein
	Pop_A08G063395	4.5497	5.08 × 10^− 16^	Hsp20/alpha crystallin family
	Pop_G17G072846	4.5303	1.35 × 10^− 18^	Hsp90 protein
	Pop_G08G057869	4.5123	1.73 × 10^− 17^	Hsp20/alpha crystallin family
	Pop_A08G063398	4.3478	8.58 × 10^− 18^	Hsp20/alpha crystallin family
	Pop_A08G063804	4.2986	3.83 × 10^− 11^	Glycosyl hydrolases family 28
	Pop_G08G058237	4.2304	7.65 × 10^− 9^	Glycosyl hydrolases family 28
	Pop_G16G016263	4.0643	1.87 × 10^− 6^	C1 domain
84K-Cg-84K-DCg-up	Pop_A06G085757	5.3306	1.31 × 10^− 28^	Nodulin-like domain
	Pop_A03G019729	3.8690	4.25 × 10^− 11^	GEM-like protein 5
	Pop_G06G075964	3.8391	7.39 × 10^− 27^	SPX domain containing protein
	Pop_G12G050744	3.5996	1.79 × 10^− 21^	Purple acid phosphatase 17
	Pop_A18G012981	3.5299	1.32 × 10^− 21^	ACT domain containing protein
	Pop_A03G034672	3.4313	2.88 × 10^− 8^	GDSL-like Lipase
	Pop_A15G014655	3.3179	1.62 × 10^− 11^	SPX domain containing protein
	Pop_A06G061615	3.2956	9.96 × 10^− 22^	Phosphodiesterase family
	Pop_A17G081472	3.1988	5.73 × 10^− 26^	Fasciclin domain
	Pop_G09G027771	3.0816	2.47 × 10^− 6^	Phosphodiesterase family
84K-Cg-84K-DCg-down	Pop_G01G089203	2.8921	1.24 × 10^− 24^	Glycine-rich protein
	Pop_G01G089192	2.8699	1.62 × 10^− 11^	Cell wall structural protein
	Pop_A06G053337	2.7789	7.89 × 10^− 22^	Heat shock protein
	Pop_A09G083657	2.4945	2.26 × 10^− 5^	FAF-like protein
	Pop_UnG071209	2.4542	7.36 × 10^− 5^	Zinc metalloprotease
	Pop_A10G046690	2.4507	2.55 × 10^− 4^	Cell wall structural protein
	Pop_G08G022160	2.4449	1.41 × 10^− 5^	Cell wall structural protein
	Pop_G06G003520	2.3571	7.75 × 10^− 6^	Heat shock protein
	Pop_A08G046066	2.3212	1.14 × 10^− 4^	Cell wall structural protein
	Pop_A14G000137	2.2370	6.76 × 10^− 4^	Lipid-binding protein

**Fig. 3:**
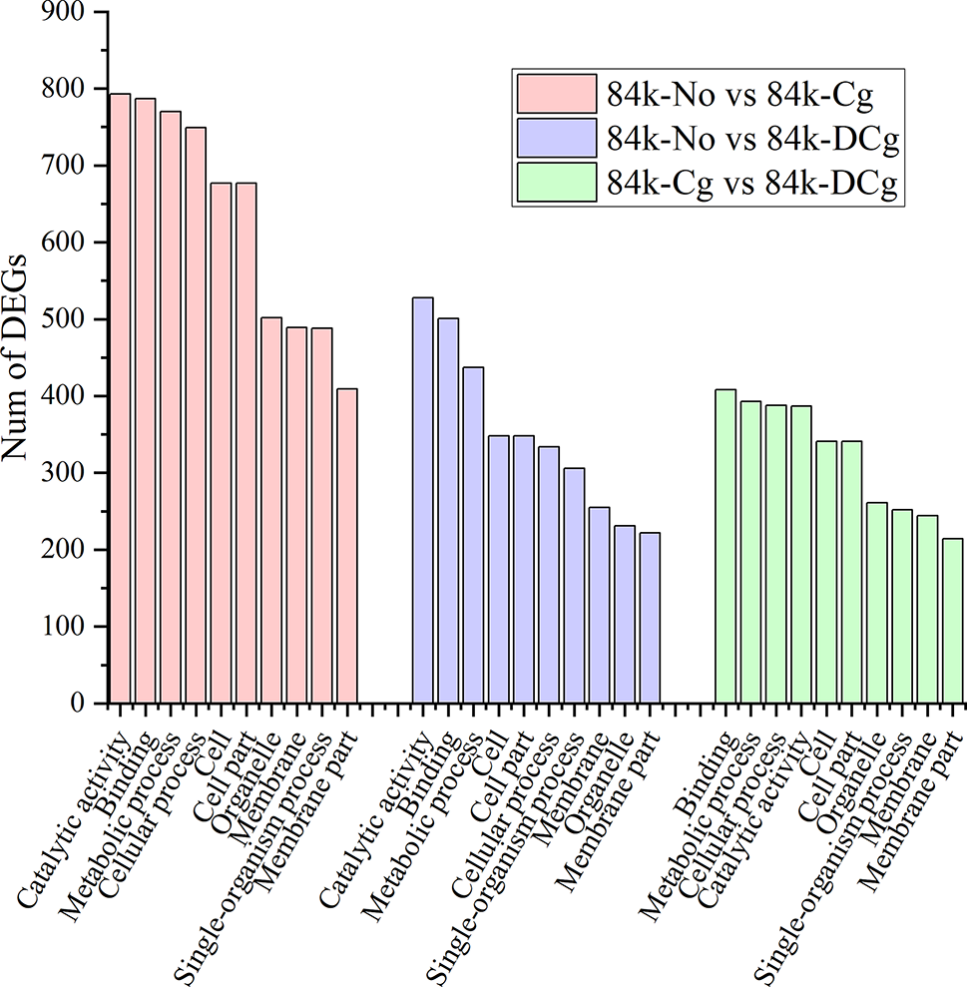
GO enrichment analysis of the DEGs

To analyze the associations between individual DEGs, we performed GO and KEGG enrichment analyses of the DEGs detected by the different comparisons. The GO enrichment analysis was performed to functionally characterize and classify all DEGs. The categories were divided into 56 subcategories. The top 10 categories in terms of the number of DEGs are presented in Fig. 3. In the biological process category, metabolic process (GO:0008152) was the most common term assigned to the DEGs. The GO analysis indicated that metabolic process was a significantly enriched GO term among the DEGs in all three comparisons (Fig. 3), of which the 84K-No vs. 84K-Cg comparison had the most DEGs (770) associated with metabolic processes, followed by the 84K-No vs. 84K-DCg comparison (437 DEGs) and the 84K-Cg vs. 84K-DCg comparison (393 DEGs).

### KEGG analysis of DEGs

**Fig. 4:**
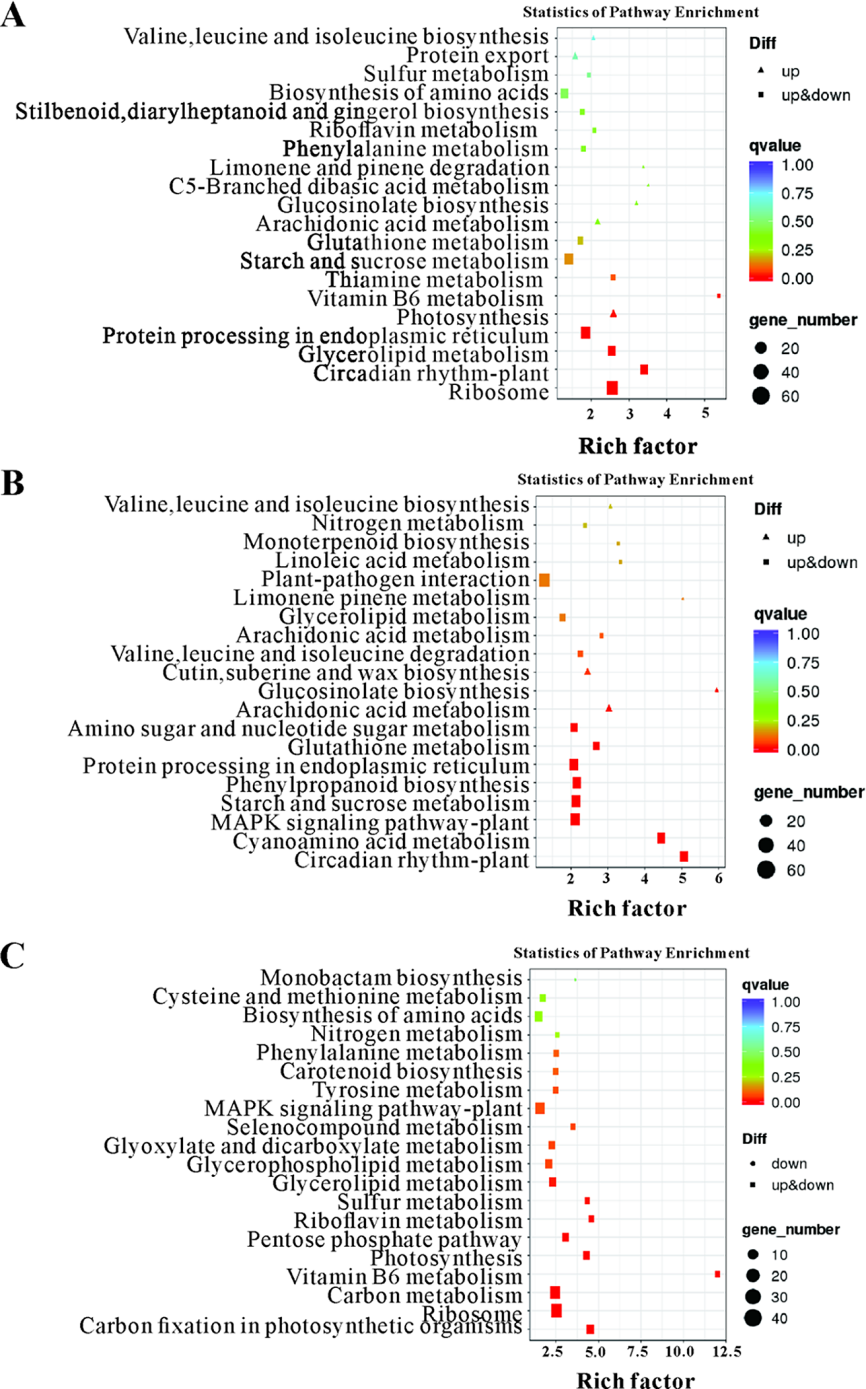
Twenty most enriched KEGG pathways among the DEGs identified by the (**A**) 84K-No vs. 84K-Cg, (**B**) 84K-No vs. 84K-DCg, and (**C**) 84K-Cg vs. 84K-DCg comparisons. The number of DEGs is reflected by the size of the circle

To further clarify the DEG functions, we conducted a KEGG enrichment analysis of all DEGs. A total of 117, 109, and 106 metabolic pathways were enriched among the DEGs detected by the 84K-No vs. 84K-Cg, 84K-No vs. 84K-DCg, and 84K-Cg vs. 84K-DCg comparisons, respectively. The 20 most enriched KEGG metabolic pathways (Fig. 4) included the following: MAPK signaling pathway–plant (ko04016), plant–pathogen interaction (ko04626), and phenylpropanoid biosynthesis (ko00940). Phenylalanine metabolism (ko00360) was not a significantly enriched KEGG pathway among the DEGs detected by the 84K-No vs. 84K-DCg comparison, but it was significantly enriched among the DEGs revealed by the 84K-No vs. 84K-Cg and 84K-Cg vs. 84K-DCg comparisons. Interestingly, the DEGs between the 84K-No and 84K-DCg samples were significantly associated with phenylpropanoid biosynthesis (ko00940). These results suggest that the 84K poplar response to a *C. gloeosporioides* infection may vary depending on the inoculation mode. Moreover, phenylpropanoid biosynthesis (ko00940) may be related to the induced resistance of 84K poplar to anthracnose.

### Verification of the expression of the genes related to phenylpropanoid biosynthesis

**Fig. 5:**
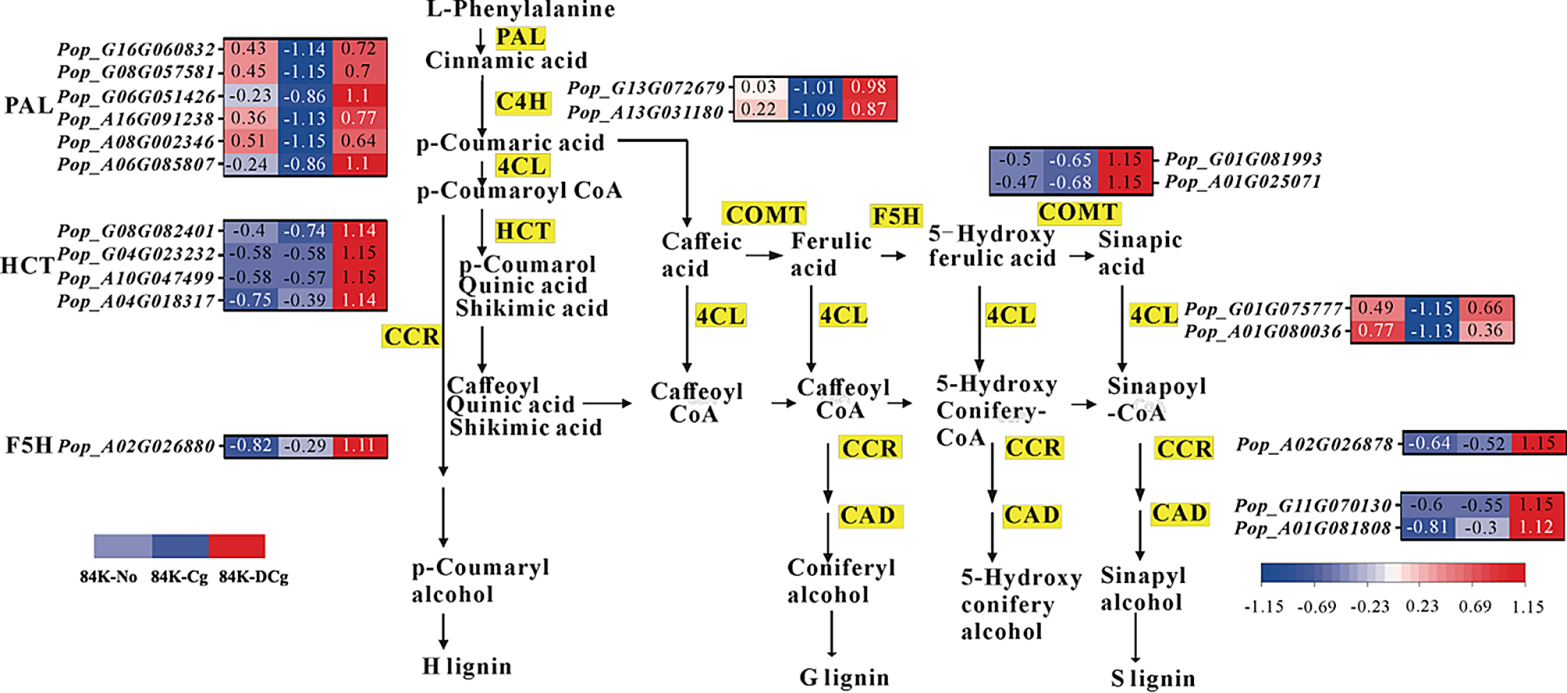
Differential expression of the structural genes in the phenylpropanoid and flavonoid biosynthesis pathways. Red and blue blocks represent upregulated and downregulated expression, respectively

Phenylpropanoid biosynthesis (ko00940) was an enriched KEGG pathway in all three groups, but not to the same extent. Thus, we analyzed the DEGs associated with this metabolic pathway in greater detail. The phenylpropanoid biosynthesis-related genes were generally more highly expressed in 84K-DCg than in 84K-No and 84K-Cg, with the lowest expression levels detected in 84K-Cg (Fig. 5). For example, the expression levels of a PAL-encoding gene (Pop_A06G085807) in 84K-No, 84K-Cg, and 84K-DCg were 276.43, 75.45, and 711.70, respectively. Accordingly, the expression level was more than 9-times higher in 84K-DCg than in 84K-Cg. Furthermore, in addition to being associated with phenylpropanoid biosynthesis (ko00940; Figure S3), this gene was also related to phenylalanine metabolism (ko00360; Figure S4). In contrast, the expression of the berberine bridge enzyme gene (Pop_A01G081808), which was also associated with phenylpropanoid biosynthesis (ko00940; Figure S3), was more similarly expressed between 84K-DCg and 84K-Cg (only 1.92-times higher in 84K-DCg than in 84K-Cg). The 16 phenylpropanoid biosynthesis-related DEGs were classified in eight gene families encoding various proteins, including PAL, cinnamate 4-hydroxylase (C4H), 4-coumaric acid-coenzyme A ligase (4CL), shikimate O-hydroxycinnamyl transferase (HCT), cinnamoyl coenzyme A reductase (CCR), caffeic acid 3-O-methyltransferase (COMT), ferulate-5-hydroxylase (F5H), and cinnamyl alcohol dehydrogenase (CAD). To verify the accuracy of the transcriptome data, we selected the phenylpropanoid biosynthesis-related DEGs in the 84K-No, 84K-Cg, and 84K-DCg samples for qRT-PCR, such as PAL, C4H, 4CL, HCT, CCR, and CAD (Figure S5). And the qRT-PCR analysis confirmed the RNA-seq data were reliable and accurate (Table S2). The enriched KEGG pathways assigned to these genes were phenylpropanoid biosynthesis (ko00940) and flavonoid biosynthesis (ko00941). For a more intuitive analysis of expression, we combined the gene expression levels and the processes associated with these two pathways (Fig. 5). Meanwhile, many phenylpropanoid and flavonoid metabolites downstream of these two pathways (ko00940 and ko00941), such as naringenin, rutin, apigenin, quercetin, catechin, and scutellarein, showed similar trends in their accumulation among different treatments, with highest concentration in the 84K-DCg samples (Table S3), which were consistent with the transcriptome results.

## Discussion

The induced resistance of a plant host activated by a local infection involves a mechanism that mediates the production of a class of chemicals and signaling molecules that are transmitted through the phloem to the uninfected parts of the plant, thereby activating various defense responses [14]. In the current study on 84K poplar inoculated with *C. gloeosporioides* spores, the lesion area was smaller and the blackening of the lesion was less extensive on the 84K-DCg leaves than on the 84K-Cg leaves at 6 days post-inoculation. Additionally, the 84K-DCg leaf surface had substantially less mycelia than the 84K-Cg leaf surface. These differences may be related to the diversity in the expression levels of defense response-related genes between 84K-DCg and 84K-Cg. The reason for the decrease in the lesion area of the 84K-DCg leaves and how induced resistance is generated were unclear. Hence, we conducted a transcriptome analysis of the 84K-No, 84K-Cg, and 84K-DCg samples to elucidate the mechanism underlying the induced resistance of 84K poplar to anthracnose.

The transcriptome analysis revealed significant differences in gene expression levels in the 84K-No, 84K-Cg, and 84K-DCg seedlings, among which 84K-No and 84K-Cg had the most DEGs, with substantial changes in the expression of various genes, especially in 84K-DCg. For example, the expression levels of the genes encoding PAL, C4H, and 4CL were significantly upregulated. Both PAL and C4H regulate the synthesis of lignin monomers, while also contributing to other pathways downstream of the phenylpropanoid metabolic pathway. These findings suggest that in 84K poplar, phenylpropanoid metabolism may be induced in response to anthracnose. Specifically, metabolic pathways associated with multiple secondary compounds, including flavonoids and lignin, may be activated. According to the results of the GO and KEGG analyses, the identified DEGs were primarily involved in metabolic processes, especially phenylpropanoid metabolism. Phenylpropanoid biosynthesis, flavonoid biosynthesis, and isoflavone biosynthesis depend on the upregulated expression of genes encoding various enzymes, including PAL, C4H, and 4CL, which affect the accumulation of cinnamic acid, coumaric acid, shikimic acid, lignin, and caffeic acid [1]. Meanwhile, many phenylpropanoid and flavonoid metabolites downstream of ko00940 (Figure S3) and ko00941 (Figure S4) pathways, such as naringenin, rutin, apigenin, quercetin, catechin, and scutellarein, showed the highest concentration in the 84K-DCg samples (Table S3), which suggested that phenylpropanoid biosynthesis (ko00940) and flavonoid biosynthesis (ko00941) may be related to the induced resistance of 84K poplar to anthracnose.

Phenylpropanoid biosynthesis is one of the most important metabolic pathways in plants. In this pathway, phenylalanine derived from the shikimate pathway is involved in reactions catalyzed by PAL, C4H, and 4CL, with the resulting metabolites used as substrates [15, 16]. Because PAL is a key inducible enzyme linking the shikimate pathway to the phenylpropanoid biosynthesis pathway, its activity and concentration directly affect the abundance of downstream secondary metabolites (e.g., lignin, flavonoids, and isoflavones). Previous research confirmed that these compounds have important physiological roles influencing plant growth and development as well as resistance to biotic and abiotic stresses [17]. The *PAL* gene family has been identified in a variety of plants, although the number of PAL-encoding genes varies among plant species. Of the nine *PAL* genes in rice, *PAL4* mediates broad-spectrum resistance to various pathogens [18]. The infection of tea plants by different pathogens leads to the significant upregulated expression of all six *PAL* genes [19]. In the present study, the phenylpropanoid and flavonoid metabolites (e.g., lignin, flavonoids, and isoflavones) were differentially expressed in the 84K-No and 84K-DCg seedlings (Table S3 and Fig. 5). Meanwhile, PAL activity and concentration directly affect the abundance of downstream phenylpropanoid and flavonoid metabolites in the 84K-DCg seedlings, such as naringenin, rutin, apigenin, quercetin, catechin, and scutellarein, which suggested that PAL-related genes may play an important role in the induced resistance of 84K poplar to anthracnose. On the other hand, some genes of 84K-Cg seedlings involved in phenylpropanoid biosynthesis such as PAL were down-regulated compared to 84K-No and 84K-DCg. It was reported that the appressorium of *C. gloeosporioides* may release effectors during infection to inhibit plant immune responses [20, 21], which may be the adaptation of *C. gloeosporioides* to host plants [12].

Compared with the corresponding expression levels in 84K-No and 84K-Cg, the genes involved in phenylpropanoid biosynthesis (ko00940) and flavonoid biosynthesis (ko00941) were more highly expressed in 84K-DCg, implying that phenylpropanoid metabolism was more active in 84K-DCg than in the other two samples. Our data also indicated that the genes encoding HCT and COMT were expressed at significantly higher levels in 84K-DCg than in 84K-No and 84K-Cg. Both HCT and COMT are considered to be the key factors controlling lignin production in the plant phenylpropanoid metabolic pathway [22]. The synthesis of lignin monomers involves a branch of the phenylpropanoid pathway. Specifically, three lignin monomers are oxidatively polymerized by laccase and peroxidase to generate high-molecular-weight lignin. In addition, lignin is tightly bound to polysaccharide molecules in the cell wall. Cross-linking forms a dense hydrophobic structure that increases the mechanical strength of plant cells and tissues, thereby acting as a natural physical barrier that inhibits the entry of pathogens [23]. There is currently considerable interest in the enzyme-catalyzed reactions in the lignin monomer synthesis pathway as well as the effects of lignin-related changes on plant immune responses, including those involving PAL, COMT, and CAD [24, 25]. In tobacco and Arabidopsis, the silencing or loss of the *PAL* gene reportedly decreases the overall immune response and increases the sensitivity to *Pseudomonas syringae* and tobacco mosaic virus [26, 27]. An earlier study demonstrated that Arabidopsis COMT mutants have enhanced mechanisms mediating responses to a variety of bacteria and fungi [28]. In the current study, the expression of genes encoding enzymes, such as PAL, COMT, HCT, and CAD, was significantly higher in 84K-DCg than in 84K-No and 84K-Cg, which may be related to the increase in the resistance of 84K-DCg to anthracnose. The improved immune response mechanism of 84K-DCg was likely critical for the observed decrease in the severity of the leaf lesions, which may be indicated phenylpropanoid biosynthesis mediated the induced resistance of 84K poplar and inproved the 84K-DCg treatments resistance to *C. gloeosporioides*.

The 84K-Cg and 84K-DCg samples were grown in the same tissue culture bottle, but they exhibited significantly different symptoms. Therefore, we hypothesized that there may be flavonoids or volatile gases in the air that directly inhibit pathogen infections or serve as a signal that stimulates an induced resistance-related mechanism. We will test this hypothesis as part of our future research. External biotic or abiotic stimuli can significantly induce the expression of plant genes related to phenylpropanoid metabolism [29], ultimately leading to a systemic immune response and the development of induced resistance.

## Conclusions

In this study, we analyzed the DEGs among the differentially inoculated 84K-No, 84K-Cg, and 84K-DCg samples using transcriptomics techniques. We subsequently focused on the DEGs significantly associated with the phenylpropanoid biosynthesis pathway. Meanwhile, PAL activity and concentration directly affect the abundance of downstream phenylpropanoid and flavonoid metabolites in the 84K-DCg seedlings, such as naringenin, rutin, apigenin, quercetin, catechin, and scutellarein, which indicated that PAL-related genes enriched in the phenylpropanoid biosynthesis may be crucial for the induced resistance of 84K poplar to anthracnose. These results will provide the basis for future research conducted to verify the contribution of phenylpropanoid biosynthesis to induced resistance and explore plant immune resistance-related signals that may regulate plant defense capabilities.

### Electronic supplementary material

Below is the link to the electronic supplementary material.

Supplementary Material 1

## Data Availability

The datasets generated during the current study are available in National Center for Biotechnology Information (NCBI) repository, accession number: PRJNA 1052950, which will be released upon publication.
