# Supplementary material for: Comparative transcriptome analysis reveals the importance of phenylpropanoid biosynthesis for the induced resistance of 84K poplar to anthracnose
Source: BMC Genomics. 2024 Mar 22;25:306. doi: 10.1186/s12864-024-10209-1 (PMC10960379; doi:10.1186/s12864-024-10209-1)
Supplement: Supplementary file 1 — Supplementary Material 1 [file 12864_2024_10209_MOESM1_ESM.docx]

Figure S1 Inoculation diagram of 84K poplar.


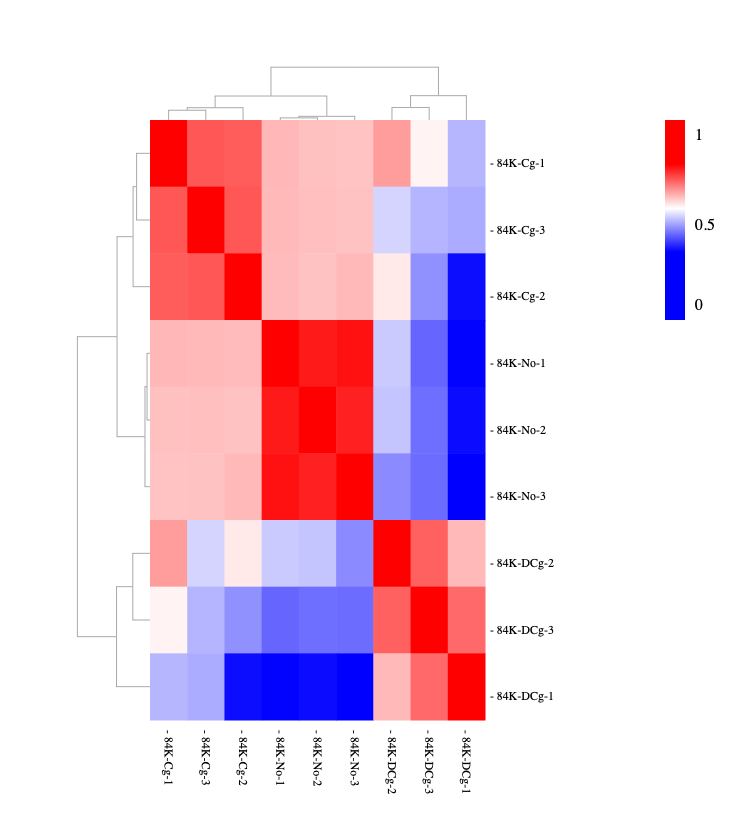


Figure S2 The heatmap of samples correlation.


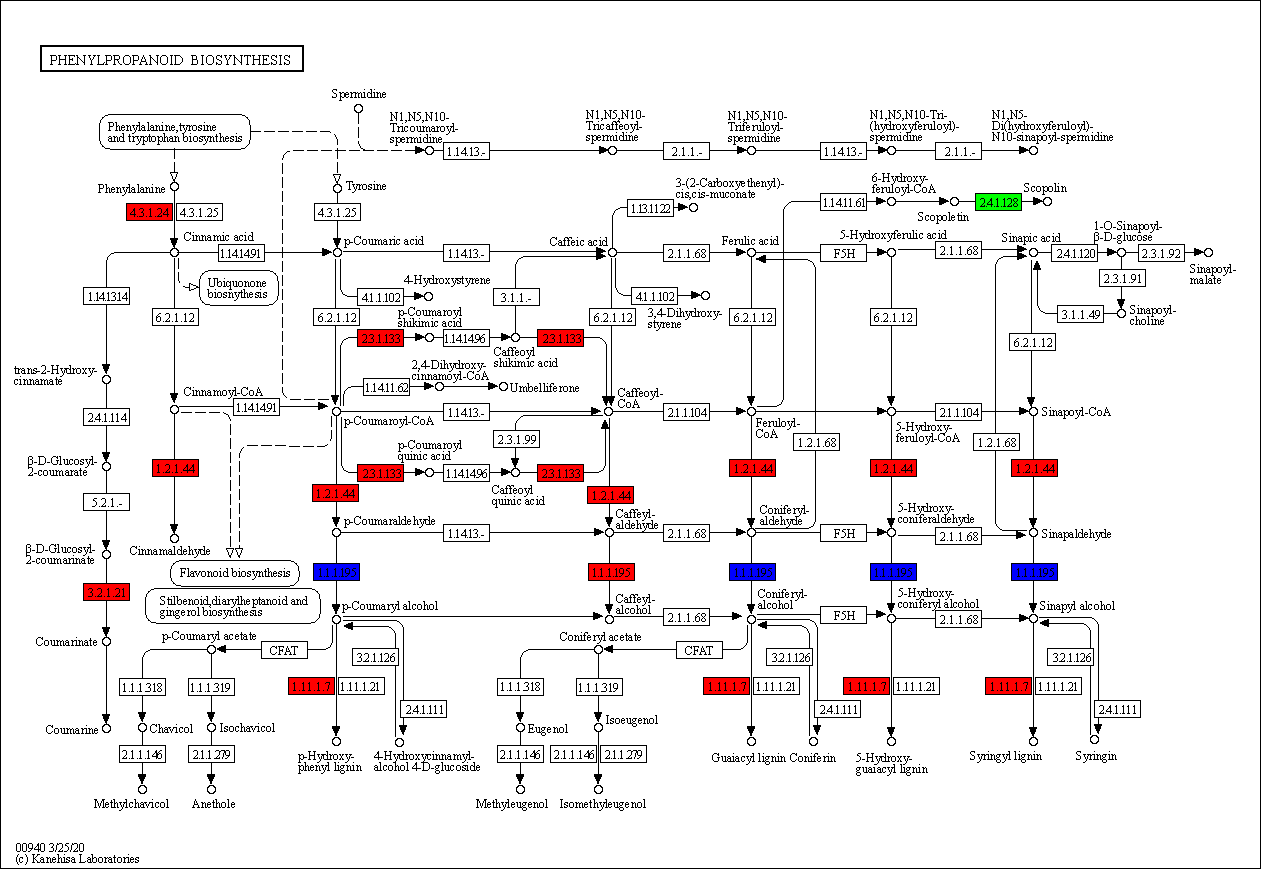


Figure S3 Phenylpropanoid biosynthesis (ko00940).


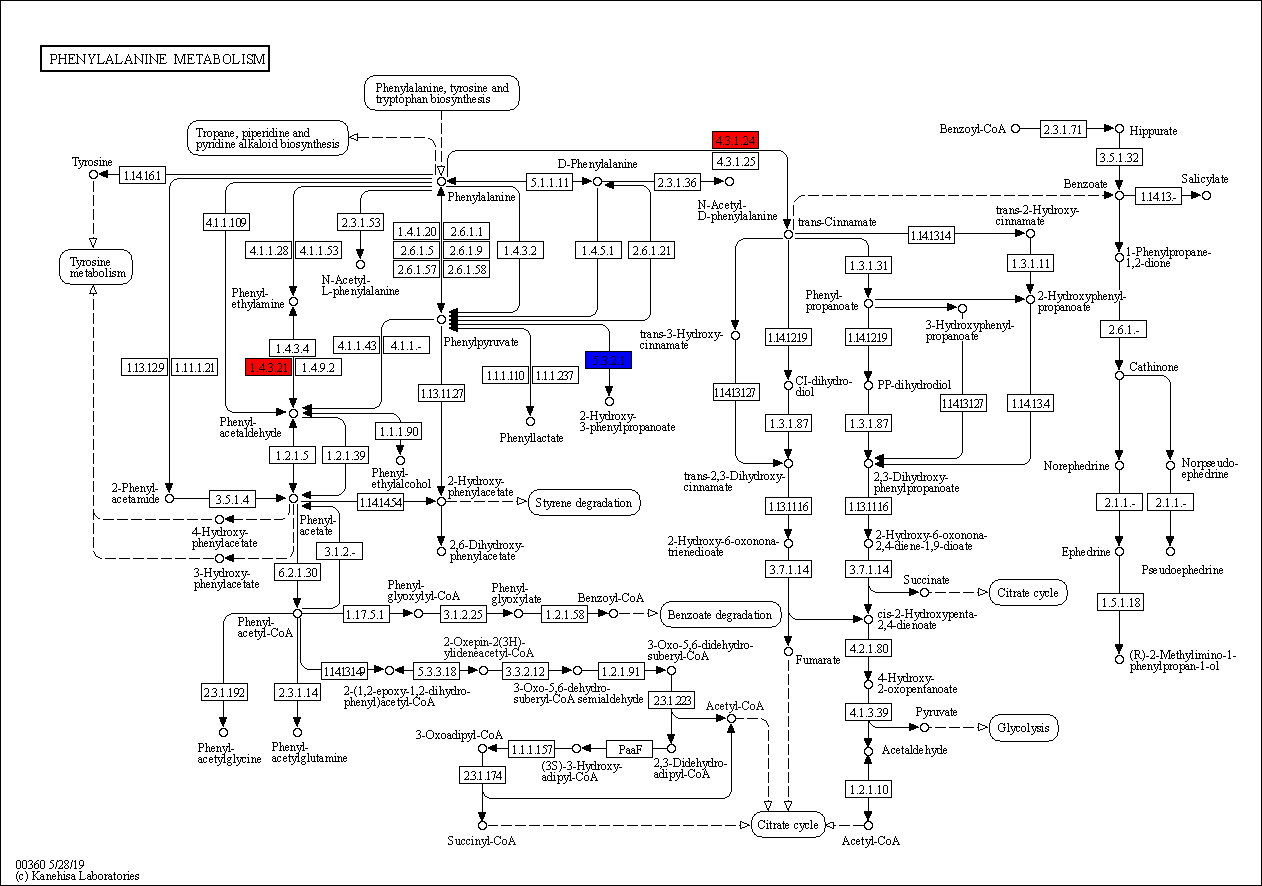


Figure S4 Phenylalanine metabolism (ko00360).


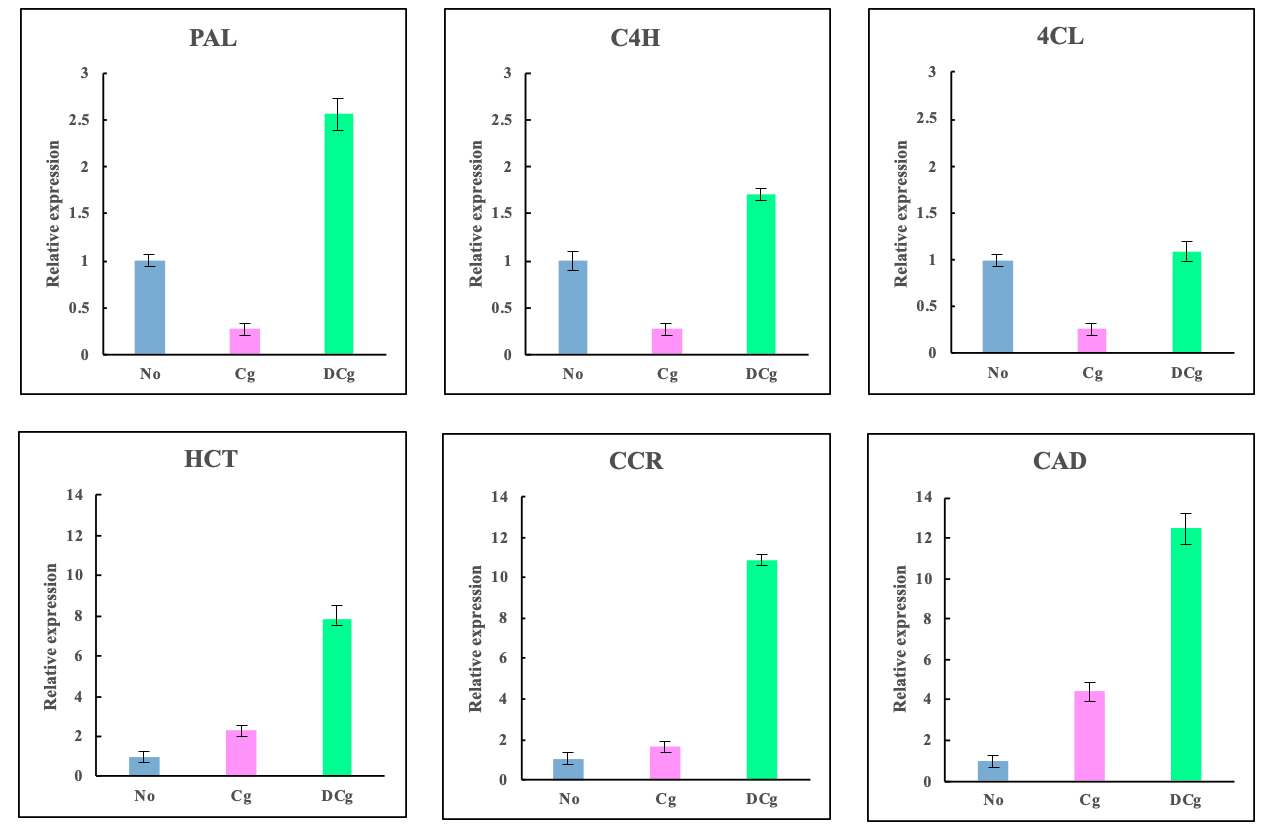


Figure S5 qRT-PCR validation of key phenylpropanoid biosynthesis-related DEGs in the 84K-No, 84K-Cg, and 84K-DCg samples. The 84K-No sample served as the control. Data from the qRT-PCR represent the mean of three replicates, and bars represent standard error.

Table S1 Primer sequences.

| Primers | 5’-3’ |
| --- | --- |
| q-18S-F | CAACCATAAACGATGCCGA |
| q-18S-R | AGCCTTGCGACCATACTCC |
| q-PAL-F | CCCTAACGTGGTTGGTGTGA |
| q-PAL-R | GGATGTGTTGGTGGGGACTT |
| q-C4H-F | TGCTCCCATCCAACCATCAC |
| q-C4H-R | CAATCCCATTGGTTGCAGCC |
| q-4CL-F | GCATCTTTAGCCCGCAATGG |
| q-4CL-R | CCTGAGGCGCTTCATTTTCG |
| q-HCT-F | AAAAGGGGGAAAGGCAACGA |
| q-HCT-R | TGGACGACGAACCTGATCAC |
| q-CCR-F | AGCTGCCAGATTAGACTGCA |
| q-CCR-R | GTGAAAAACCCCGGCACATC |
| q-CAD-F | ACGCACTGTGGAGTTTGCTA |
| q-CAD-R | AGGCCCTGAAATACGGCAAA |

Table S2 The FPKM values of key phenylpropanoid biosynthesis-related DEGs in the 84K-No, 84K-Cg, and 84K-DCg samples.

| Gene | #ID | 84K-  No-1 | 84K-  No-2 | 84K-  No-3 | 84K-  Cg-1 | 84K-  Cg-2 | 84K-  Cg-3 | 84K-  DCg-1 | 84K-  DCg-2 | 84K-  DCg-3 |
| --- | --- | --- | --- | --- | --- | --- | --- | --- | --- | --- |
| PAL | Pop_A06G085807 | 276.433624 | 270.469021 | 279.451327 | 79.215824 | 75.447762 | 67.331793 | 711.701233 | 782.46582 | 797.083531 |
| C4H | Pop_G13G072679 | 17.564608 | 18.893324 | 18.228966 | 4.815556 | 3.825146 | 4.320351 | 30.103136 | 5.41685 | 17.759993 |
| HCT | Pop_A04G018317 | 2.251438 | 2.354431 | 2.302935 | 15.09376 | 5.179471 | 10.136616 | 17.700315 | 13.919952 | 15.810134 |
| 4CL | Pop_G01G075777 | 19.107372 | 19.000345 | 19.053859 | 5.190397 | 3.683831 | 4.437114 | 20.791649 | 3.725528 | 12.258589 |
| CCR | Pop_A02G026878 | 1.703148 | 1.365778 | 1.534463 | 7.811796 | 2.846047 | 5.328922 | 18.578611 | 13.17695 | 15.877814 |
| CAD | Pop_G11G070130 | 0.099745 | 0.100057 | 0.098674 | 0.445412 | 0.208404 | 0.326908 | 4.378907 | 1.247616 | 2.813262 |

Table S3 Main differences in the phenylpropanoid and flavonoid of different treatments as determined by HPLC-MS/MS

| Name | Formula | Class | 84K-  No-1  （μg/mL） | 84K-  No-2  （μg/mL） | 84K-  No-3  （μg/mL） | 84K-  Cg-1  （μg/mL） | 84K-  Cg-2  （μg/mL） | 84K-  Cg-3  （μg/mL） | 84K-  DCg-1  （μg/mL） | 84K-  DCg-2  （μg/mL） | 84K-  DCg-3  （μg/mL） |
| --- | --- | --- | --- | --- | --- | --- | --- | --- | --- | --- | --- |
| Scutellarein 6,7,4'-trimethyl ether 5-glucoside | C_24_H_26_O_11_ | Flavonoids | 98.472 | 96.593 | 100.536 | 136.043 | 135.125 | 132.854 | 377.679 | 376.343 | 364.454 |
| 7-[3,4-dihydroxy-5-(hydroxymethyl)oxolan-2-yl]oxy-2-(4-hydroxyphenyl)-3,4-dihydro-2H-chromene-3,5-diol | C_20_H_22_O_9_ | Flavonoids | 98.950 | 97.677 | 101.483 | 53.608 | 52.349 | 55.236 | 88.677 | 85.543 | 91.843 |
| 6,3',4'-Trihydroxy-4-methoxy-5-methylaurone | C_17_H_14_O_6_ | Flavonoids | 2.843 | 2.861 | 2.799 | 25.533 | 25.893 | 24.581 | 86.522 | 87.764 | 83.234 |
| Naringenin | C_15_H_12_O_5_ | Flavonoids | 57.464 | 56.543 | 54.670 | 46.295 | 45.305 | 45.235 | 104.568 | 102.024 | 103.654 |
| Chrysin | C_15_H_10_O_4_ | Flavonoids | 56.666 | 57.476 | 59.742 | 34.155 | 35.021 | 34.822 | 62.877 | 65.098 | 62.134 |
| Genistein | C_15_H_10_O_5_ | Flavonoids | 23.968 | 23.058 | 23.444 | 29.035 | 29.315 | 28.698 | 77.653 | 79.546 | 77.000 |
| 4,2'-Dihydroxy-4',6'-dimethoxychalcone 4-glucoside | C_23_H_26_O_10_ | Flavonoids | 38.088 | 37.678 | 38.876 | 10.987 | 11.025 | 11.263 | 0.367 | 0.908 | 0.546 |
| Vitexin 2"-O-p-coumarate | C_30_H_26_O_12_ | Flavonoids | 0.144 | 0.123 | 0.147 | 0.085 | 0.082 | 0.084 | 0.155 | 0.165 | 0.147 |
| isorhamnetin-3-O-glucoside | C_22_H_22_O_12_ | Flavonoids | 48.777 | 46.754 | 49.011 | 14.241 | 13.799 | 14.352 | 1.067 | 1.543 | 1.221 |
| Rutin | C_27_H_30_O_16_ | Flavonoids | 4.544 | 4.879 | 4.777 | 7.511 | 7.638 | 7.527 | 21.743 | 21.855 | 21.567 |
| 7-Hydroxy-2-(4-hydroxyphenyl)-4-oxo-3,4-dihydro-2H-chromen-5-yl β-D-glucopyranoside | C_21_H_22_O_10_ | Flavonoids | 4.548 | 4.700 | 4.675 | 4.795 | 5.085 | 5.053 | 12.234 | 13.097 | 13.011 |
| apigetrin | C_21_H_20_O_10_ | Flavonoids | 3.333 | 3.915 | 3.098 | 2.686 | 2.900 | 2.895 | 6.067 | 6.235 | 7.033 |
| Glycitein | C_16_H_12_O_5_ | Flavonoids | 7.678 | 7.555 | 7.269 | 3.447 | 3.467 | 3.366 | 4.387 | 4.580 | 4.512 |
| (2S,3S,4S,5R,6S)-6-[2-(3,4-dihydroxyphenyl)-5-hydroxy-4-oxochromen-7-yl]oxy-3,4,5-trihydroxyoxane-2-carboxylic acid | C_21_H_18_O_12_ | Flavonoids | 2.433 | 2.540 | 2.776 | 3.803 | 3.646 | 3.821 | 10.876 | 10.222 | 10.597 |
| apigenin-7-O-glucuronide | C_21_H_18_O_11_ | Flavonoids | 3.170 | 3.204 | 3.180 | 2.559 | 2.631 | 2.549 | 5.788 | 6.004 | 5.743 |
| Catechin-1 | C_15_H_14_O_6_ | Flavonoids | 8.666 | 8.489 | 8.233 | 3.044 | 2.937 | 2.857 | 1.987 | 1.790 | 1.765 |
| isorhamnetin-3-O-rutinoside | C_28_H_32_O_16_ | Flavonoids | 1.434 | 1.678 | 1.555 | 2.867 | 2.865 | 2.965 | 8.600 | 8.349 | 8.822 |
| Isorhamnetin | C_16_H_12_O_7_ | Flavonoids | 7.000 | 7.458 | 7.638 | 2.635 | 2.917 | 3.017 | 2.222 | 2.753 | 2.922 |
| 7-hydroxy-2-[4-[(2S,3R,4S,5S,6R)-3,4,5-trihydroxy-6-(hydroxymethyl)oxan-2-yl]oxyphenyl]-2,3-dihydrochromen-4-one | C_21_H_22_O_9_ | Flavonoids | 4.876 | 4.987 | 4.766 | 1.682 | 1.851 | 1.721 | 1.010 | 1.490 | 1.256 |
| Quercetin-3β-D-glucoside | C_21_H_20_O_12_ | Flavonoids | 6.666 | 6.773 | 6.497 | 2.637 | 2.715 | 2.545 | 2.563 | 2.728 | 2.412 |
| 2-(3,4-Dihydroxyphenyl)-5-hydroxy-7-methoxy-4-oxo-4H-chromen-3-yl 2-O-β-D-xylopyranosyl-β-D-glucopyranoside | C_27_H_30_O_16_ | Flavonoids | 5.765 | 5.809 | 5.622 | 2.057 | 2.079 | 2.063 | 1.434 | 1.467 | 1.600 |
| (2S,3S,4S,5R,6S)-3,4,5-trihydroxy-6-[5-hydroxy-2-(4-hydroxyphenyl)-6-methoxy-4-oxochromen-7-yl]-oxyoxane-2-carboxylic acid | C_22_H_20_O_12_ | Flavonoids | 1.521 | 1.266 | 1.654 | 2.723 | 2.749 | 2.873 | 8.011 | 8.355 | 8.401 |
| Kaempferol-3-O-rutinoside | C_27_H_30_O_15_ | Flavonoids | 1.566 | 1.523 | 1.707 | 1.320 | 1.483 | 1.606 | 3.054 | 3.666 | 3.915 |
| Luteolin | C_15_H_10_O_6_ | Flavonoids | 2.099 | 2.200 | 2.411 | 1.351 | 1.288 | 1.390 | 2.631 | 2.309 | 2.455 |
| (2R,3R)-2-(3,4-dihydroxyphenyl)-3,5-dihydroxy-7-methoxy-2,3-dihydrochromen-4-one | C_16_H_14_O_7_ | Flavonoids | 4.510 | 4.713 | 4.822 | 1.587 | 1.644 | 1.686 | 1.043 | 1.041 | 1.078 |
| (2S,3S,4S,5R,6S)-3,4,5-trihydroxy-6-(5-hydroxy-4-oxo-2-phenylchromen-7-yl)oxyoxane-2-carboxylic acid | C_21_H_18_O_10_ | Flavonoids | 0.349 | 0.380 | 0.403 | 1.046 | 1.014 | 1.235 | 3.311 | 3.169 | 3.919 |
| Astragalin | C_21_H_20_O_11_ | Flavonoids | 4.466 | 4.289 | 4.453 | 1.670 | 1.698 | 1.732 | 1.378 | 1.655 | 1.610 |
| Apigenin-7-O-neohesperidoside | C_27_H_30_O_14_ | Flavonoids | 1.888 | 1.765 | 1.599 | 1.765 | 1.907 | 1.786 | 4.289 | 4.911 | 4.653 |
| 4',5,7-trihydroxy-3,6-dimethoxyflavone | C_17_H_14_O_7_ | Flavonoids | 5.158 | 5.578 | 5.464 | 1.788 | 1.886 | 1.867 | 1.099 | 1.023 | 1.072 |
| Quercetin | C_15_H_10_O_7_ | Flavonoids | 2.664 | 2.205 | 2.118 | 1.618 | 1.281 | 1.367 | 2.998 | 2.279 | 2.665 |
| 3-Methoxy-5,7,3',4'-tetrahydroxy-flavone | C_16_H_12_O_7_ | Flavonoids | 0.775 | 0.848 | 0.632 | 0.497 | 0.479 | 0.438 | 0.965 | 0.830 | 0.900 |
| 2-(3,5-dihydroxy-4-methoxyphenyl)-5,7-dihydroxy-3-[(2S,3R,4R,5R,6S)-3,4,5-trihydroxy-6-methyloxan-2-yl]oxychromen-4-one | C_22_H_22_O_12_ | Flavonoids | 1.012 | 1.056 | 1.020 | 0.603 | 0.604 | 0.599 | 1.099 | 1.059 | 1.076 |
| (2R,3R)-3,5-dihydroxy-2-(4-hydroxyphenyl)-7-methoxy-2,3-dihydrochromen-4-one | C_16_H_14_O_6_ | Flavonoids | 0.443 | 0.587 | 0.489 | 0.939 | 0.984 | 0.753 | 2.844 | 2.856 | 2.146 |
| Hispidulin | C_16_H_12_O_6_ | Flavonoids | 1.388 | 1.448 | 1.454 | 0.539 | 0.573 | 0.604 | 0.499 | 0.559 | 0.659 |
| taxifolin | C_15_H_12_O_7_ | Flavonoids | 1.765 | 1.055 | 1.651 | 1.040 | 0.635 | 0.807 | 1.876 | 1.166 | 1.172 |
| 2,4,6-Trihydroxy-2-(4-hydroxybenzyl)-1-benzofuran-3(2H)-one | C_15_H_12_O_6_ | Flavonoids | 0.555 | 0.498 | 0.523 | 0.437 | 0.419 | 0.435 | 0.973 | 0.969 | 0.998 |
| Quercetin 3-O-malonylglucoside | C_24_H_22_O_15_ | Flavonoids | 0.976 | 0.905 | 0.999 | 0.285 | 0.267 | 0.293 | 0.023 | 0.030 | 0.027 |
| Formononetin | C_16_H_12_O_4_ | Flavonoids | 0.870 | 0.800 | 0.800 | 0.376 | 0.356 | 0.372 | 0.447 | 0.445 | 0.503 |
| (2Z)-4,6-dihydroxy-2-[(4-hydroxy-3,5-dimethoxyphenyl)methylidene]-1-benzofuran-3-one | C_17_H_14_O_7_ | Flavonoids | 0.089 | 0.068 | 0.085 | 0.029 | 0.024 | 0.027 | 0.011 | 0.015 | 0.009 |
| Kaempferol 3-glucuronide | C_21_H_18_O_12_ | Flavonoids | 1.506 | 1.545 | 1.532 | 0.543 | 0.555 | 0.548 | 0.394 | 0.399 | 0.386 |
| Catechin-2 | C_15_H_14_O_6_ | Flavonoids | 0.111 | 0.143 | 0.139 | 0.759 | 0.777 | 0.783 | 2.545 | 2.576 | 2.601 |
